# Childhood solid tumours in relation to population mixing around the time of birth

**DOI:** 10.1038/sj.bjc.6600880

**Published:** 2003-04-29

**Authors:** T A Nyari, H O Dickinson, D M Hammal, L Parker

**Affiliations:** 1 North of England Children's Cancer Research Unit, Department of Child Health, University of Newcastle, Royal Victoria Infirmary, Queen Victoria Road, Newcastle upon Tyne NE1 4LP, UK

**Keywords:** childhood solid tumours, population mixing, epidemiology

## Abstract

In a retrospective cohort study of 673 787 live births in the Northern Region of England, 1975–1994, we investigated whether a higher level of population mixing around birth was a risk factor for solid tumours, by diagnostic group (Hodgkin's disease, brain and spinal tumours, neuroblastoma, other solid tumours), diagnosed during 1975–2001 under age 15 years. Logistic regression was used to relate risk to population mixing, based on (i) all movers and (ii) incomers from outside the region. Both ward and county district level analyses were performed. There was a decreased risk of brain and spinal tumours with increasing population mixing based on incomers from outside the region (OR for trend across three categories=0.79, 95% CI: 0.66–0.95, *P*=0.01 in the ward level analysis). Although this may be because of chance, it is consistent with a role of exposure to infection and immunological response in the aetiology of these tumours. For other tumour groups, there was no consistent evidence of an association between risk and population mixing.

A series of studies by Kinlen and others has demonstrated an association between the risk of childhood leukaemia and population mixing, which has been interpreted as evidence of an infectious aetiology (Kinlen, 1995; Alexander *et al*, 1997). However, few studies have investigated the possible association between other childhood malignancies and population mixing (Cartwright *et al*, 2001). In earlier studies of children born in the county of Cumbria, northwest England, we found no association between the risk of all solid tumours and population mixing (Dickinson and Parker, 1999). However, we subsequently reported an association between exposure around birth to high levels of population mixing and a lower risk of brain and spinal tumours and a possible association between parental migration and a higher risk of neuroblastoma (Dickinson *et al*, 2002b). Recent epidemiological studies have suggested that exposure to infections before or around birth may be associated with the risk of brain tumours in children (Linet *et al*, 1996; Linos *et al*, 1998; McKinney *et al*, 1999; Fear *et al*, 2001; McNally *et al*, 2002). In addition, it has been suggested that JC virus has a role in the aetiology of brain tumours (Khalili, 2001). Further, there is substantial evidence that exposure to infections may be a risk factor for Hodgkin's disease, especially in children (Douglas *et al*, 1998; Stiller, 1998).

The aim of the current study was to investigate in a much larger cohort and a different geographical area from our previous studies (Dickinson and Parker, 1999; Dickinson *et al*, 2002b), whether levels of population mixing around the time of birth were a risk factor for solid tumours, both overall and by diagnostic group, in children aged 0–14 years.

## METHODS

### Study area

The area considered was the Northern Region of England, as defined by the 1972 Boundary Commission (Local Government Act, 1972), excluding the county of Cumbria (which has about 14% of the annual total of around 35 000 births in the Northern Region), as the relation between population mixing around birth and risk of solid tumours for children born there has already been considered in separate analyses (Dickinson and Parker, 1999; Dickinson *et al*, 2002b). Children born during 1975–1994 were considered. Births and cases were assigned to the 507 wards (median area=3.6 km^2^) and 23 county districts (median area=186 km^2^), as defined in the 1981 census, within the study area.

### Ascertainment of cases

Registrations of first malignancies for children, born and diagnosed under age 15 years in the Northern Region before the end of 2001, were obtained from the Northern Region Young Person's Malignant Disease Registry (Cotterill *et al*, 2000). We excluded retinoblastoma, as almost half the cases are hereditary (Narod *et al*, 1991); gender-specific tumours, as the population at risk differs; cases born in Cumbria; and nine cases for whom no birth information was available. Cases were assigned to diagnostic groups, as shown in Table 1

**Table 1:** Number of cases and incidence (per 100 000 person-years) of solid tumours by diagnostic group, among children born in Northern Region (excluding Cumbria) during 1975–1994 and diagnosed before the end of 2001 (rounding off may result in total incidence differing from the sum of incidence by diagnostic group)

**Age (years)**	**0–4**	**5–14**	**0–14**			
**Total person-years**	**3 368 935**		**5 673 451**		**9 042 386**	
**Diagnostic group**	**No. of cases**	**Incidence**	**No. of cases**	**Incidence**	**No. of cases**	**Incidence**
*All solid tumours*	306	9.1	306	5.4	612	6.8
Hodgkin's disease	4	0.1	40	0.7	44	0.5
Brain and spinal tumours	105	3.1	139	2.5	244	2.7
Neuroblastoma	64	1.9	12	0.2	76	0.8
Other solid tumours	133	3.9	115	2.0	248	2.7

, using a standard classification for childhood cancer (Birch and Marsden, 1987; Kramarova and Stiller, 1996). These diagnostic groups were selected to correspond to those for which there has been some evidence of an association with infections or migration; other tumours were grouped together. Postcodes of birth addresses of cases were assigned to wards and county districts (MIMAS, 2002).

### Birth population

The dates and county districts of birth of all live birth registrations in the Northern Region in the study period were obtained from the Regional Office of the Department of Health; hence, the numbers of births in each county district in each year were calculated. For births during 1981–1994, the postcode of birth was also available and births were assigned to wards (MIMAS, 2002) and the numbers of births in each ward in each year were likewise calculated. The numbers of births in each ward in each year during 1975–1980 were estimated by assuming that, within each county district, the proportion of births in each ward during 1975–1980 was the same as during 1981–1994.

### Population mixing

The total numbers of residents (OPCS, 1981a; ONS, 1991), all movers (those who had changed address in the year before the census) and incomers from outside the region (OPCS, 1981b; ONS, 1991) were extracted from 1981 and 1991 census data and aggregated to 1981 wards and county districts. Since about 20% of 1981 wards changed in the 1991 census, data from the 1991 census were obtained for enumeration districts (which are subareas within wards) and aggregated to 1981 wards (MIMAS, 2001).

Numbers of residents and movers within county districts in each noncensus year during 1975–1994 were estimated by linear interpolation or extrapolation. Numbers within wards were estimated by linear interpolation for intercensal years. Before 1981 extrapolation at ward level resulted in a few implausible estimates, so numbers within wards were assumed to be the same proportion of the county district estimates as in the nearest census. We also carried out an analysis restricted to children born during 1981–1994, in order to assess the sensitivity of the results to potential errors introduced by this estimate of the population during 1975–1980.

Measures of migration were then calculated for each ward and each county district: (i) the proportion of all movers and (ii) the proportion of incomers from outside the region; each measure was grouped such that one-quarter of the births were in a low group, one-half in a medium group and one-quarter in a high group.

### Statistical methods

Analysis was carried out for all children under 15 years and for the age groups 0–4 and 5–14 years. Hodgkin's disease was not considered in the age group 0–4 years as there were only four cases.

Logistic regression was used to investigate the relation between risk of solid tumours (overall and within each diagnostic group) and each measure of population mixing (Hosmer and Lemeshow, 1989). Odds ratios (OR) and 95% confidence intervals (CI), derived from a quadratic approximation to the log likelihood, are reported. Odds ratios correspond to a trend in risk across the low, medium and high groups giving each group equal weight.

## RESULTS

The ranges of population mixing as percent, as in Table 2

**Table 2:** Distribution of percentage of migrants in wards and county districts

	**Wards**		**County districts**	
	**Median**	**(Lowest, highest)**	**Median**	**(Lowest, highest)**
*Northern region (excluding Cumbria)*				
Incomers from outside the region	0.9	(0.05, 29)	1.0	(0.3, 3.4)
All migrants	8.4	(3.1, 38)	8.7	(6.9, 11)
				
*England and Wales (Dickinson* etal*, 2002a)*				
Incomers from outside the region	1.5	(0.0, 54)	1.8	(1, 4)
All migrants	8.5	(3.1, 38)	9.2	(7, 12)

, for wards based on incomers from outside the regions were 0.05–0.6, 0.6–1.5 and 1.5–29 in the low, medium and high categories respectively; and based on all migrants were 3.1–7.0, 7.0–10.0 and 10.0–38, respectively.

The ranges of population mixing for county districts based on incomers from outside the regions were 0.3–0.8, 0.8–1.6 and 1.6–3.4 in the low, medium and high categories, respectively; and based on all migrants were 6.9–8.2, 8.2–9.4 and 9.4–11.0, respectively.

There were 673 787 live births in the study area, during 1975–1994; 8% of these were in rural wards. There was insufficient statistical power to perform an analysis considering urban/rural status as a covariate.

Table 1 shows the numbers of cases and incidence of solid tumours by age and diagnostic group.

### Pattern of population mixing

The pattern of migration in the study area was compared with that in England and Wales, using data from a previous study (see Table 2) (Dickinson *et al*, 2002a). As expected, the distribution was very skewed especially for incomers from outside the region, with a few wards having proportions of movers several times the median. There was a much wider range of population mixing at ward level than at county district level, reflecting considerable heterogeneity between wards within county districts. Although the median proportion of all movers was similar in the study area and in England and Wales, the range at ward level in the study area was restricted. The proportion of incomers from outside the region was substantially lower in the study area than in England and Wales. The median population mixing in Seascale, both for all migrants and for incomers from outside the region, was higher than that observed in the rest of Cumbria or in the remainder of the Northern Region. However, both Cumbria and the remainder of the Northern Region included wards with more extreme levels of population mixing than observed in Seascale, although the population – in particular the child population – in these wards tended to be low.

As expected, in the study area the proportion of all movers was highly correlated with the proportion of incomers from outside the region: *ρ*=0.62 and 0.43 (*P*<0.001 and *P*=0.04, respectively) at ward and county district level, respectively.

### Risk of solid tumours in relation to population mixing (see Table 3

**Table 3:** Odds ratios for risk of solid tumours by age and diagnostic group in relation to population mixing at ward and county district level

		**County district level**			**Ward level**		
**Diagnostic group**	**Population mixing based on**	**OR**	**95% Cl**	***P***	**OR**	**95% Cl**	***P***
**(A) Age 0–14 years**							
*All solid tumours*	Incomers from outside the region	**0.88**	**(0.79–0.98)**	**0.03**	**0.84**	**(0.75–0.94)**	**0.003**
	All migrants	1.02	(0.91–1.14)	0.75	0.92	(0.82–1.03)	0.15
Hodgkin's disease	Incomers from outside the region	**0.60**	**(0.39–0.92)**	**0.02**	**0.57**	**(0.37–0.88)**	**0.01**
	All migrants	0.94	(0.62–1.44)	0.79	0.76	(0.50–1.16)	0.20
Brain and spinal tumours	Incomers from outside the region	0.87	(0.73–1.04)	0.12	**0.79**	**(0.66–0.95)**	**0.01**
	All migrants	0.98	(0.82–1.17)	0.83	0.85	(0.71–1.02)	0.08
Other solid tumours^a^	Incomers from outside the region	0.98	(0.82–1.17)	0.85	0.97	(0.81–1.15)	0.72
	All migrants	1.10	(0.92–1.31)	0.30	0.99	(0.83–1.18)	0.93
							
**(B) Age 0–4 years**							
*All solid tumours*	Incomers from outside the region	0.97	(0.83–1.13)	0.68	0.92	(0.78–1.08)	0.29
	All migrants	0.90	(0.76–1.05)	0.18	1.05	(0.90–1.23)	0.52
Brain and spinal tumours	Incomers from outside the region	0.98	(0.75–1.29)	0.89	0.84	(0.64–1.10)	0.21
	All migrants	0.9	(0.68–1.18)	0.44	0.98	(0.75–1.29)	0.89
Neuroblastoma	Incomers from outside the region	0.73	(0.51–1.04)	0.08	0.85	(0.60–1.21)	0.38
	All migrants	0.79	(0.56–1.12)	0.19	1.10	(0.78–1.55)	0.60
Other solid tumors^b^	Incomers from outside the region	1.09	(0.86–1.39)	0.47	1.05	(0.82–1.33)	0.72
	All migrants	0.93	(0.73–1.18)	0.56	1.06	(0.83–1.35)	0.63
							
**(C) Age 5–14 years**							
*All solid tumours*	Incomers from outside the region	**0.80**	**(0.68–0.94)**	**0.01**	**0.77**	**(0.66–0.91)**	**0.002**
	All migrants	1.16	(0.99–1.36)	0.07	**0.80**	**(0.69–0.94)**	**0.01**
Hodgkin's disease	Incomers from outside the region	**0.57**	**(0.36–0.90)**	**0.01**	**0.60**	**(0.38–0.94)**	**0.02**
	All migrants	0.89	(0.58–1.39)	0.62	0.67	(0.43–1.04)	0.07
Brain and spinal tumours	Incomers from outside the region	0.79	(0.63–1.00)	0.05	**0.76**	**(0.60–0.96)**	**0.02**
	All migrants	1.05	(0.83–1.33)	0.7	**0.77**	**(0.61–0.98)**	**0.03**
Other solid tumours^a^	Incomers from outside the region	0.87	(0.67–1.13)	0.29	0.88	(0.68–1.15)	0.35
	All migrants	**1.33**	**(1.03–1.73)**	**0.03**	0.92	(0.71–1.19)	0.51

Odds ratios are for a trend in risk across the three equally weighted categories of population mixing. Significant effects (*P*<0.05) are shown in bold.

a Excludes neuroblastoma.

b Excludes Hodgkin's disease.

)Among children aged 0–14 years (see Table 3A), the county district level analysis showed that the risk of all solid tumours decreased significantly with increasing population mixing, based on incomers from outside the region (OR=0.88, 95% CI: 0.79–0.98, *P*=0.03); this effect was determined largely by the significantly decreasing risk of Hodgkin's disease with increasing population mixing (OR=0.60, 95% CI: 0.39–0.92, *P*=0.02), but partly also by a nonsignificantly decreasing risk of brain and spinal tumours (OR=0.87, 95% CI: 0.73–1.04, *P*=0.12). The ward level analysis showed similar results, but the decreasing risk of brain and spinal tumours with increasing population mixing was significant (OR=0.79, 95% CI: 0.66–0.95, *P*=0.01). There were no associations with population mixing based on all movers. Among children aged 0–4 years (see Table 3B), neither the county district nor the ward-level analysis showed any significant association with population mixing.

Among children aged 5–14 years (see Table 3C), both the county district and ward level analyses showed that the risk of all solid tumours decreased significantly with increasing population mixing, based on incomers from outside the region. This effect was consistent for all tumour groups and was significant in the ward-level analysis for brain and spinal tumours and Hodgkin's disease (OR=0.76, 95% CI: 0.60–0.96, *P*=0.02, and OR=0.60, 95% CI: 0.38–0.94, *P*=0.02, respectively). In the county district level analysis, the associations were broadly similar. For population mixing based on all movers, the results at ward level were similar to those for incomers from outside the region, but at county district level, there were no significant associations except for an increased risk of other solid tumours with increasing population mixing; this was an isolated finding and is likely to be due to chance.

Restriction of the analysis to children born during 1981–1994 gave similar results for brain and spinal tumours, but the associations between population mixing and risk of Hodgkin's disease were no longer significant.

## DISCUSSION

### Strengths and weaknesses of study

The Northern Region Young Person's Malignant Disease Registry estimate that they register over 98% of cases of childhood cancer diagnosed in the Northern Region and the diagnoses of all registered cases are reviewed centrally (Cotterill *et al*, 2000). Cases born in the Northern Region but diagnosed elsewhere were not ascertained, which could lead to underestimation of the effects of population mixing. However, we believe the number of such cases to be very small since the incidence in each diagnostic group in the birth cohort was very similar to that reported elsewhere for the Northern Region (Cotterill *et al*, 2000). The proportion of cases diagnosed outside the Northern Region is likely to be less than 5%, since a previous study of childhood leukaemia among children born in the region estimated that only 24 out of 447 (5%) were diagnosed elsewhere (Parker *et al*, 1998). We considered two measures of population mixing – one based on all movers and one on movers from greater distances – as the latter has been found to be a greater risk factor for childhood leukaemia (Stiller and Boyle, 1996; Dickinson *et al*, 2002a). We performed analyses at two levels of areal aggregation, allowing assessment of whether heterogeneity within the larger areas obscured any associations of exposure with risk.

Previous analyses of risk of solid tumours in relation to ward level population mixing were based on a cohort of children born in Cumbria, northwest England (Dickinson and Parker, 1999; Dickinson *et al*, 2002b); these studies differed from the present study in that they used a different definition of population mixing (proportion of parents in a ward who were born outside the county), considered population mixing as a continuous variable, and had different age and diagnostic groups. These earlier studies did not consider population mixing based on incomers from outside the region, which we found to be more significantly associated with risk than population mixing based on all movers. However, these studies ascertained cases of cancer among children born in the study area but diagnosed elsewhere. Analysis for children born in Cumbria using the current measure of population mixing yielded results broadly consistent with the current study for the only comparable groups: all solid tumours, brain and spinal tumours, and other solid tumours in the younger age group, analysed at ward level. Therefore, any differences between the findings of the current study and our study of risk of solid tumours in Cumbrian-born children (Dickinson *et al*, 2002b) are likely to be due to the different definitions of population mixing.

#### All solid tumours

Our earlier studies found no significant variation in risk of all solid tumours with increasing population mixing (OR for trend from lowest to highest observed level of population mixing=1.2, 95% CI: 0.3–4.0 (Dickinson and Parker, 1999) and OR=0.9, 95% CI: 0.2–2.9 (Dickinson *et al*, 2002b)). This is consistent with the findings of the present study (OR for trend over three categories=0.92, 95% CI: 0.82–1.03 for population mixing based on all movers). However, when we considered population mixing based on incomers from outside the region, the present study found a significant association (OR=0.84, 95% CI: 0.75–0.94).

#### Brain and spinal tumours

Our finding of a significantly decreased risk of brain and spinal tumours in relation to ward level population mixing based on incomers from outside the region, especially among older children, may be a chance finding subsequent to multiple hypothesis testing. However, this finding was consistent with our earlier findings of a nonsignificantly decreasing risk with increasing population mixing, in particular, a decreased risk among children whose parents were migrants (Dickinson *et al*, 2002b). Other studies have suggested that neonatal exposure to infections is a risk factor for brain tumours, especially for astrocytomas and ependymomas in the younger age group (McNally *et al*, 2002). Although marked rural population mixing has generally been followed by increases of linked malignancies, it is possible that largely urban mixing, as here, may produce reduced incidence if transmission of lower (and therefore immunising) doses of the relevant agent was promoted because of the higher population density and higher background immunity, as suggested for childhood leukaemia in overspill new towns (Kinlen *et al*, 1990).

#### Hodgkin's disease

Our earlier investigations of Hodgkin's disease found no significant variation in risk with community population mixing (Dickinson *et al*, 2002b). While the present study found a decreased risk with increasing population mixing for children born during 1975–1994, this finding was not confirmed by a sensitivity analysis restricted to children born during 1981–1994.

#### Neuroblastoma

In our earlier study of Cumbrian children, subset analysis showed an increased risk of neuroblastoma among children of incomers (Dickinson *et al*, 2002b). We therefore considered neuroblastoma as a separate group in the present analysis, in order to test the hypothesis that population mixing was a risk factor for this tumour. However, our current findings provide no evidence that population mixing is associated with the risk of neuroblastoma.

#### Other solid tumours

Our earlier investigations of these tumours (which included neuroblastoma) showed a significantly increased risk among children of incomers in the age group 0–6 years (Dickinson *et al*, 2002b). We found no significant variation in risk of these tumours with population mixing in the younger age group in the present study.

### Conclusion

The risk of brain and spinal tumours was higher among children born in areas with a lower proportion of incomers from outside the region. Studies in other populations are needed to investigate whether this observed association is because of chance. As population mixing can only be a surrogate for the actual risk factor, further studies should consider other putative exposures and individual characteristics, which may reflect susceptibility. There is no consistent evidence that population mixing is associated with the risk of neuroblastoma or other solid tumours. The greater level of significance in the county district compared to the ward level analyses highlight the need to perform ecological studies using small areal units.
